# Combining bioinformatics, network pharmacology and artificial intelligence to predict the mechanism of celastrol in the treatment of type 2 diabetes

**DOI:** 10.3389/fendo.2022.1030278

**Published:** 2022-10-19

**Authors:** Ming Wu, Yan Zhang

**Affiliations:** ^1^ Postgraduate Training Base in Shanghai Gongli Hospital, Ningxia Medical University, Shanghai, China; ^2^ Department of Orthopedics, Gongli Hospital of Pudong New Area, Shanghai, China

**Keywords:** bioinformatics, type 2 diabetes, network pharmacology, celastrol, AlphaFold2, molecular docking

## Abstract

**Background:**

Type 2 diabetes (T2D) is a common chronic disease with many serious complications. Celastrol can prevent and treat type 2 diabetes by reversing insulin resistance in a number of ways. However, the specific mechanisms by which celastrol prevents and treats T2D are not well understood. The aim of this study was to explore the key gene targets and potential signaling pathway mechanisms of celastrol for the treatment of T2D.

**Methods:**

GSE184050 was downloaded from the Gene Expression Omnibus online database. Blood samples from patients and healthy individuals with T2D were analyzed to identify differentially expressed genes (DEGs), and a protein−protein interaction network (PPI) was constructed. Key gene analysis of DEGs was performed using the MCODE plugin in Cystoscope as well as the Hubba plugin, and intersections were taken to obtain hub genes, which were displayed using a Venn diagram. Enrichment analysis was then performed *via* the ClueGo plugin in Cytoscape and validated using Gene Set Enrichment Analysis. The therapeutic targets of celastrol were then analyzed by pharmacophore network pharmacology, intersected to identify the therapeutic targets of celastrol, enriched for all targets, and intersected to obtain the signaling pathways for celastrol treatment. The protein structures of the therapeutic targets were predicted using the artificial intelligence AlphaFold2. Finally, molecular docking was used to verify whether celastrol could be successfully docked to the predicted targets.

**Results:**

618 DEGs were obtained, and 9 hub genes for T2D were identified by the MCODE and Hubba plug-ins, including ADAMTS15, ADAMTS7, ADAMTSL1, SEMA5B, ADAMTS8, THBS2, HBB, HBD and HBG2. The DEG-enriched signaling pathways mainly included the ferroptosis and TGF-beta signaling pathways. A total of 228 target genes were annotated by pharmacophore target analysis, and the therapeutic targets were identified, including S100A11, RBP3, HBB, BMP7 and IQUB, and 9 therapeutic signaling pathways were obtained by an intersectional set. The protein structures of the therapeutic targets were successfully predicted by AlphaFold2, and docking was validated using molecular docking.

**Conclusion:**

Celastrol may prevent and treat T2D through key target genes, such as HBB, as well as signaling pathways, such as the TGF-beta signaling pathway and type II diabetes mellitus.

## Introduction

Type 2 diabetes (T2D) is a chronic disease with a high prevalence worldwide (1), and there is no effective drug to cure T2D (2). Many serious complications of T2D can pose a threat to human health (3). Obesity is a very common complication (4), and excessive obesity can put the bones under great stress (5). Patients with T2D are prone to osteoporosis, which further increases the probability of fractures (6). High blood glucose levels can lead to the development of atherosclerotic cardiovascular disease in the blood vessels, affecting the quality and duration of patient survival (7).

The Gene Expression Omnibus (GEO) (8) is the most commonly used public database containing sequencing data from a large number of disease tissue samples (8). Bioinformatics (9) is used for gene expression profiling to further investigate the molecular mechanisms and signaling pathways of disease through differentially expressed gene (DEG) screening (10). Analysis of key genes (11) and signaling pathways (12) in the development of disease can help lead to better cures (13).

Chinese medicine has been an important part of clinical practice in China for thousands of years. Numerous studies have shown the effectiveness of Chinese medicine in the treatment of various chronic diseases with minimal side effects (14). Celastrol is a natural phytochemical (15) and plays important roles in the regulation of blood sugar (16) and in the treatment of T2D (17). Network pharmacology is a common method of drug therapeutic target analysis, through which the therapeutic target genes of a drug can be identified (18). There have been many reports using bioinformatics and network pharmacology approaches to shed light on the use of celastrol for the treatment of diabetes and its complications, such as insulin resistance (19), diabetic nephropathy (20) and diabetic cardiomyopathy (21). The structure of a protein determines its function of the protein, and new therapeutic drugs can be developed based on this structure (22). AlphaFold2 (23) is a novel biocomputational technology that greatly reduces the difficulty of drug development by accurately predicting protein structures (24).

In this study, we first screened the blood samples for DEGs using the Limma package and obtained the hub genes using 2 key gene analysis methods to obtain intersections. GO and KEGG enrichment of DEGs was performed to identify the signaling pathways enriched by DEGs, and the results were further validated using Gene Set Enrichment Analysis (GSEA) (25). Pharmacophore network pharmacology was used to analyze the gene targets of celastrol treatment. Enrichment analysis of pharmacophore targets was performed to identify potential signaling pathways for the treatment of T2D. Molecular dynamics validation was achieved using AlphaFold2 and molecular docking. The results of these experiments provide new insights into the potential gene targets and signaling pathways for the treatment of T2D with celastrol.

## Materials and methods

### Data collection and study design

**Figure 1:**
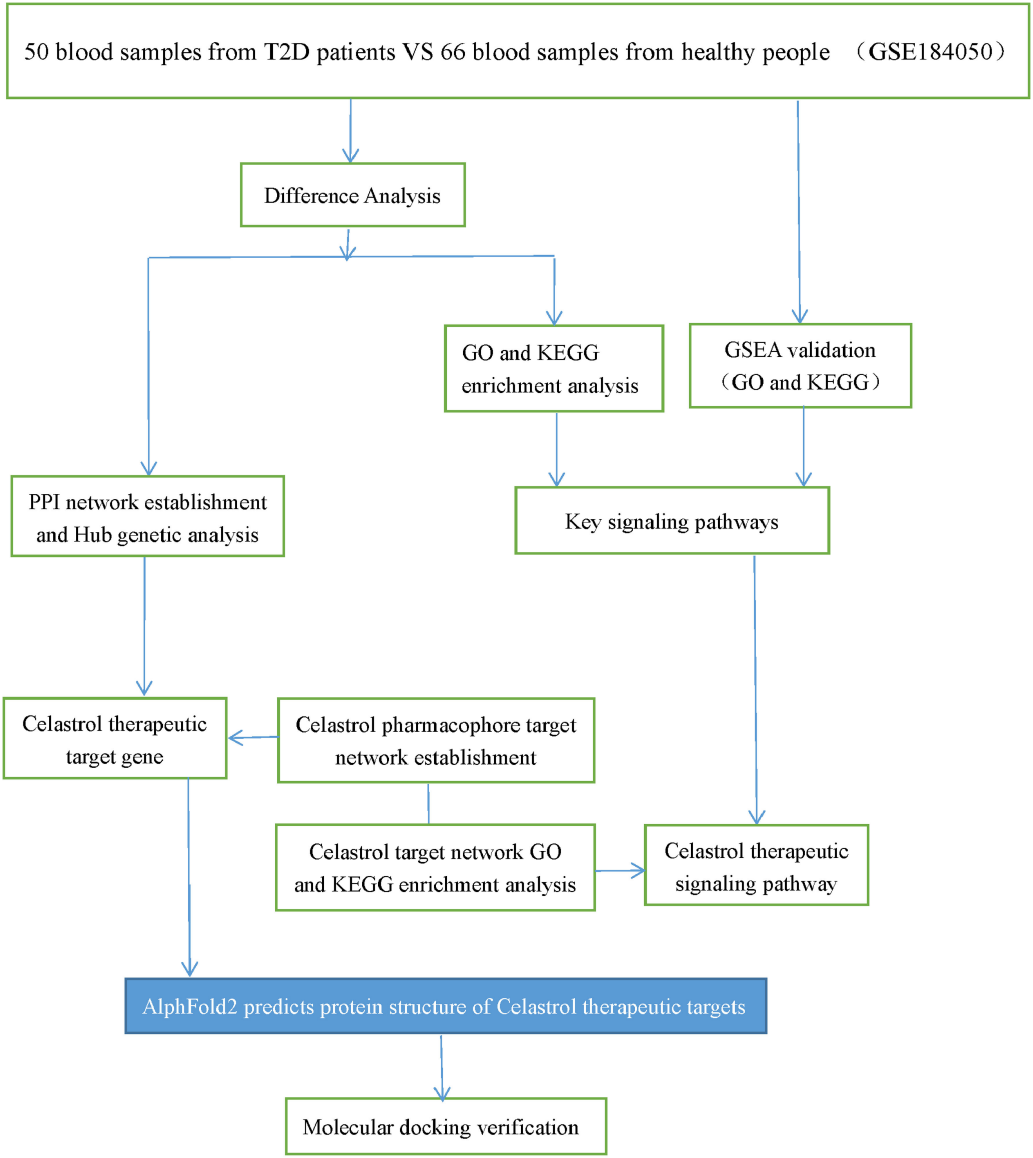
Study design flow chart. Schematic representation of the analysis carried out in this study.

The GEO database is the largest comprehensive online database of gene expression, and the GSE184050 dataset was identified for download (26). The dataset contained 116 samples, 50 blood samples from T2D patients and 66 blood samples from healthy individuals. The flow chart of the study design is shown in **Figure 1**.

### Identification of differentially expressed genes

First, two groups of samples were grouped according to the GSE184050 dataset, and analysis of variance was carried out in R (4.1.0) software using the “Limma” package, which is a commonly used analysis of variance package. Thresholds of |Log2(Fold Change)| > 0.5, p < 0.05 were used as screening conditions to identify significant DEGs.

### Identification of hub genes

The STRING online database is a database for predicting protein−protein interactions. “Multiple protein” was selected from the STRING online database (27), and the DEGs were uploaded to the database to generate protein interaction networks (PPIs) and visualized using Cytoscape tools. The CytoHubba plug-in was used to identify the central genes, the MCODE plug-in was used to analyze the important module genes, and the key genes identified by the two methods were intersected to obtain the hub genes.

### Enrichment analysis of differentially expressed genes

Cytoscape is a software program that graphically displays networks and performs analysis and editing. The ClueGo plug-in for Cytoscape (v3.9.0) (28) was used for enrichment analysis in Gene Ontology (GO) and contained annotations for Biological Process (BP), Cell Composition (CC) and Molecular Function (MF), and Kyoto Protocol Encyclopedia of Genes and Genomes (KEGG) pathway analysis was performed to plot them into diagrams.

### Gene set enrichment analysis

The 116 samples were divided into two groups, diabetic blood samples and healthy blood samples, which were analyzed for GO and KEGG enrichment by GSEA to enrich all genes expressed in the samples. The corresponding datasets were set up from the Molecular Signature Database. The T2D-related signaling pathways were validated against the enrichment results, and the validation results were graphically presented.

### Celastrol pharmacophore target network construction

The top 600 highest scores were used as screening criteria to predict celastrol targets using the pharmMapper database (29), the most widely used online database for drug target prediction based on pharmacophore analysis. In the UniProt database (30), the drug target gene species was selected as “Homo sapiens”, and a drug target network was created in Cytoscape based on drug target relationships.

### Construction of a network target for celastrol

The 228 target genes successfully analyzed by pharmacophore analysis and annotation were subjected to GO and KEGG enrichment analyses using Cytoscape’s ClueGo plugin to obtain the target signaling pathway for celastrol, which was then intersected with the signaling pathway enriched by DEGs and displayed using a Venn diagram to obtain the signaling pathway for celastrol in T2D.

### Protein structure prediction using AlphaFold2

AlphaFold2 is an artificial intelligence program that predicts protein structures with an accuracy that is at the level of predictions observed by humans using sophisticated instruments such as cryoelectron microscopy.

Five therapeutic target genes were obtained by taking the intersection of target genes of celastrol and DEGs of T2D, and the protein codes of the human species corresponding to the target genes were downloaded from the UniProt database (30), which is the most informative and widely available protein database. The protein codes were then used to perform an online structure prediction search in AlphaFold2 and download a PDB format file of the corresponding protein structure for a display map of the protein structure.

### Molecular docking

The 2D structure SDF file of celastrol was downloaded from the PubChem database as a small-molecule ligand drug. The receptor was hydrogenated by AutoDocktools software (31) to convert it into PDBQT. It was then converted into a 2D structure to find the active pocket of the protein. Finally, the molecules were docked using AutoDockvina software, and the minimum free energy was selected for visualization.

### Statistical analysis

R (4.1.0) was used for bioinformatics analysis, and the R package was used for statistical analysis. P < 0.05 was considered statistically significant.

## Results

### Identification of 618 differentially expressed genes in type 2 diabetes

**Figure 2:**
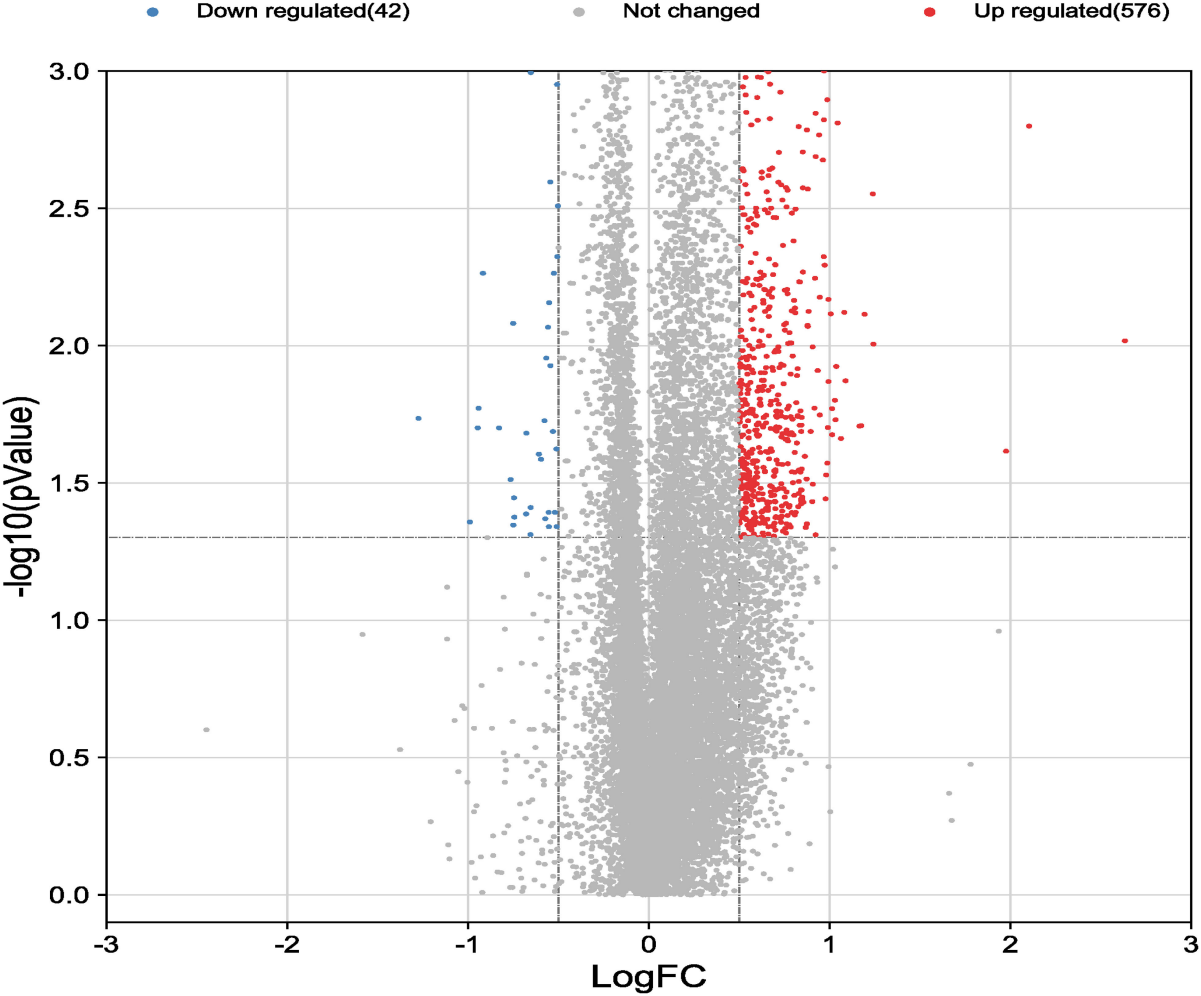
Volcano map of differentially expressed genes. Volcano map of all genes detected in the GSE184050 dataset. Each dot represents a gene. The dashed lines delineate the regions of downregulated and upregulated genes. The screening criteria for significant genes were |log2FC| > 0.5 and P < 0.05.

**Figure 3:**
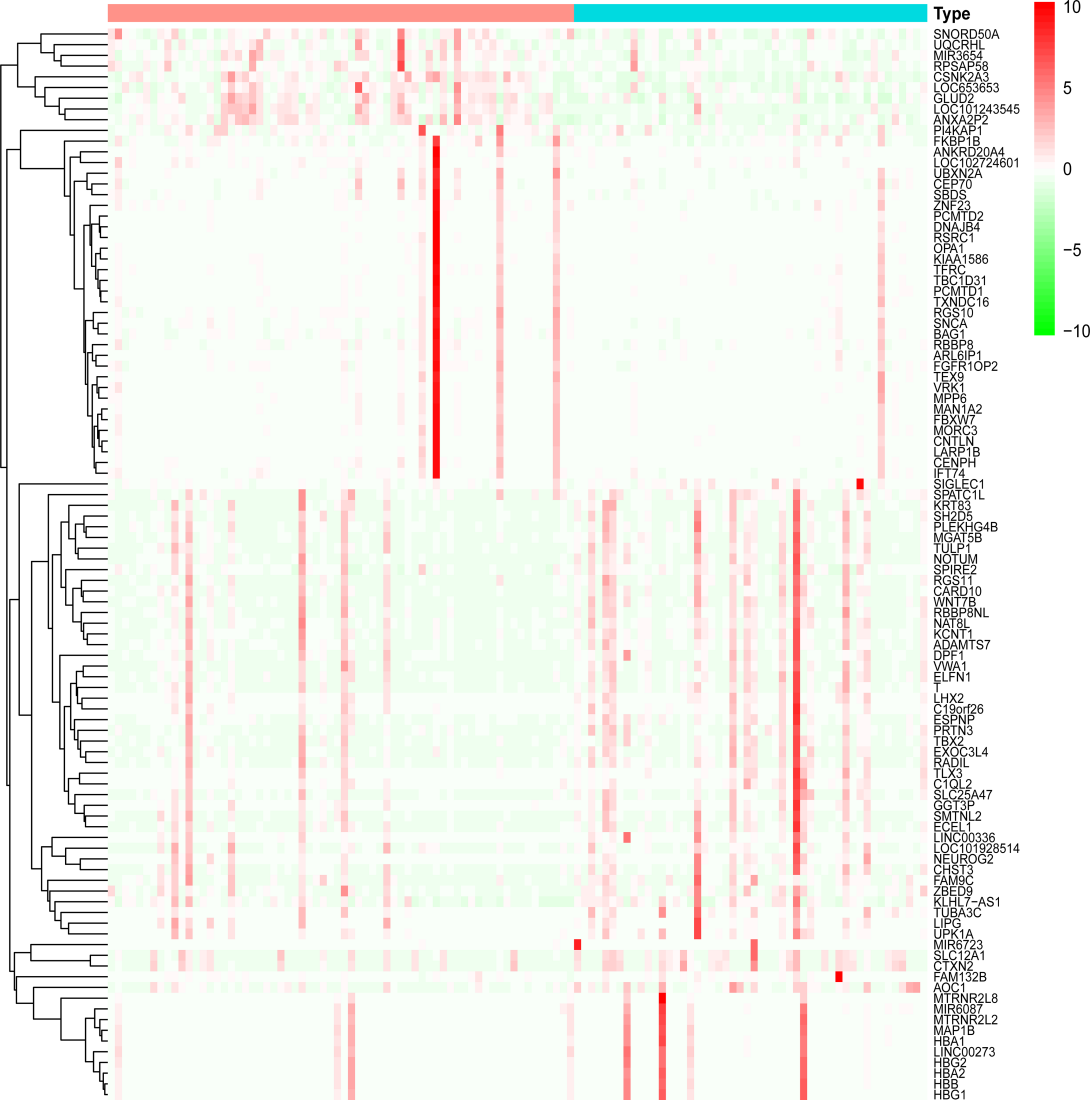
Heatmap of differentially expressed genes. Heatmap display of the clustering of differentially expressed genes (DEGs). Red indicates the group of healthy human blood samples, and blue indicates the group of type 2 diabetes blood samples.

**Figure 4:**
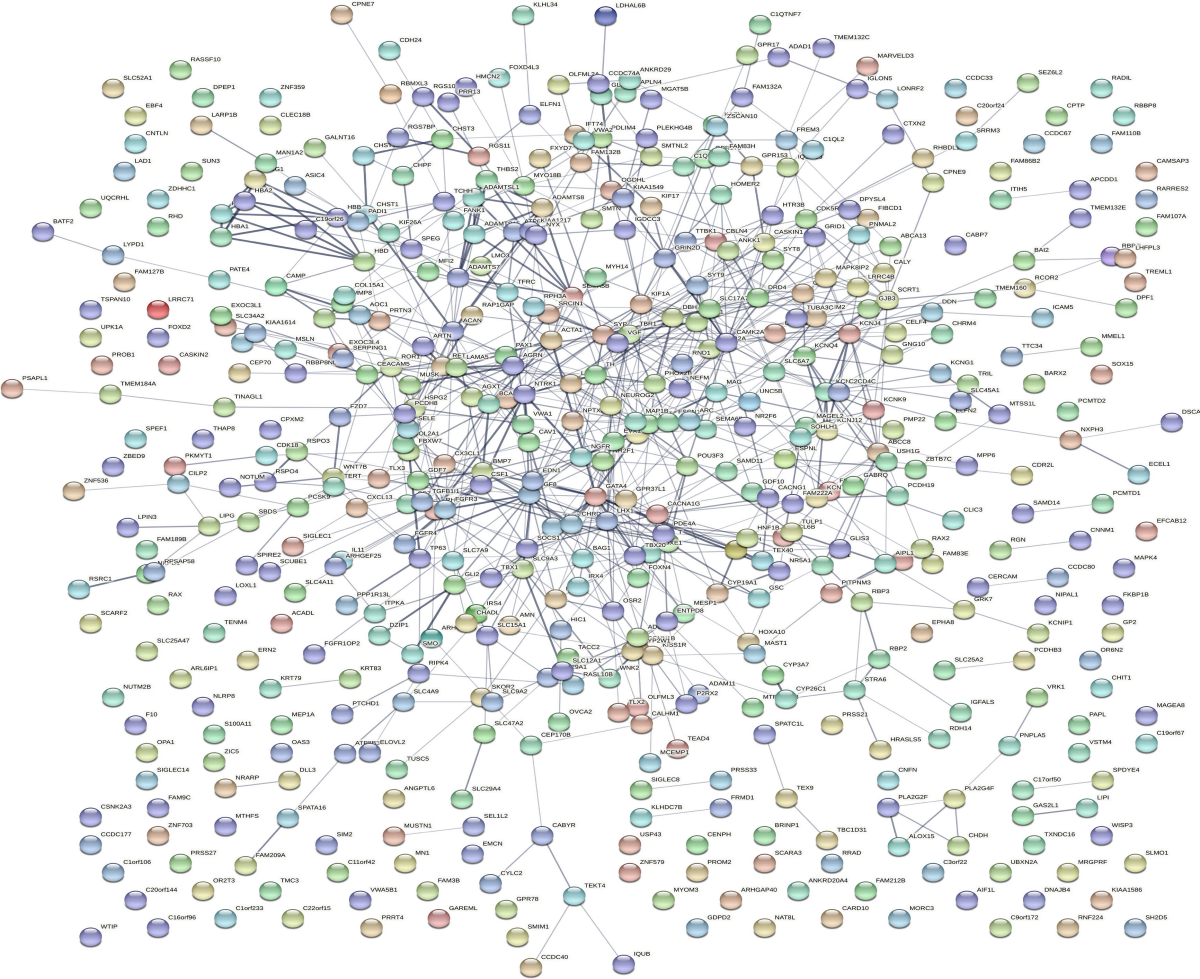
Protein interaction networks of differentially expressed genes. Protein−protein interaction network map of 618 DEGs obtained from the online database STRING. Each orb represents a differential gene.

**Figure 5:**
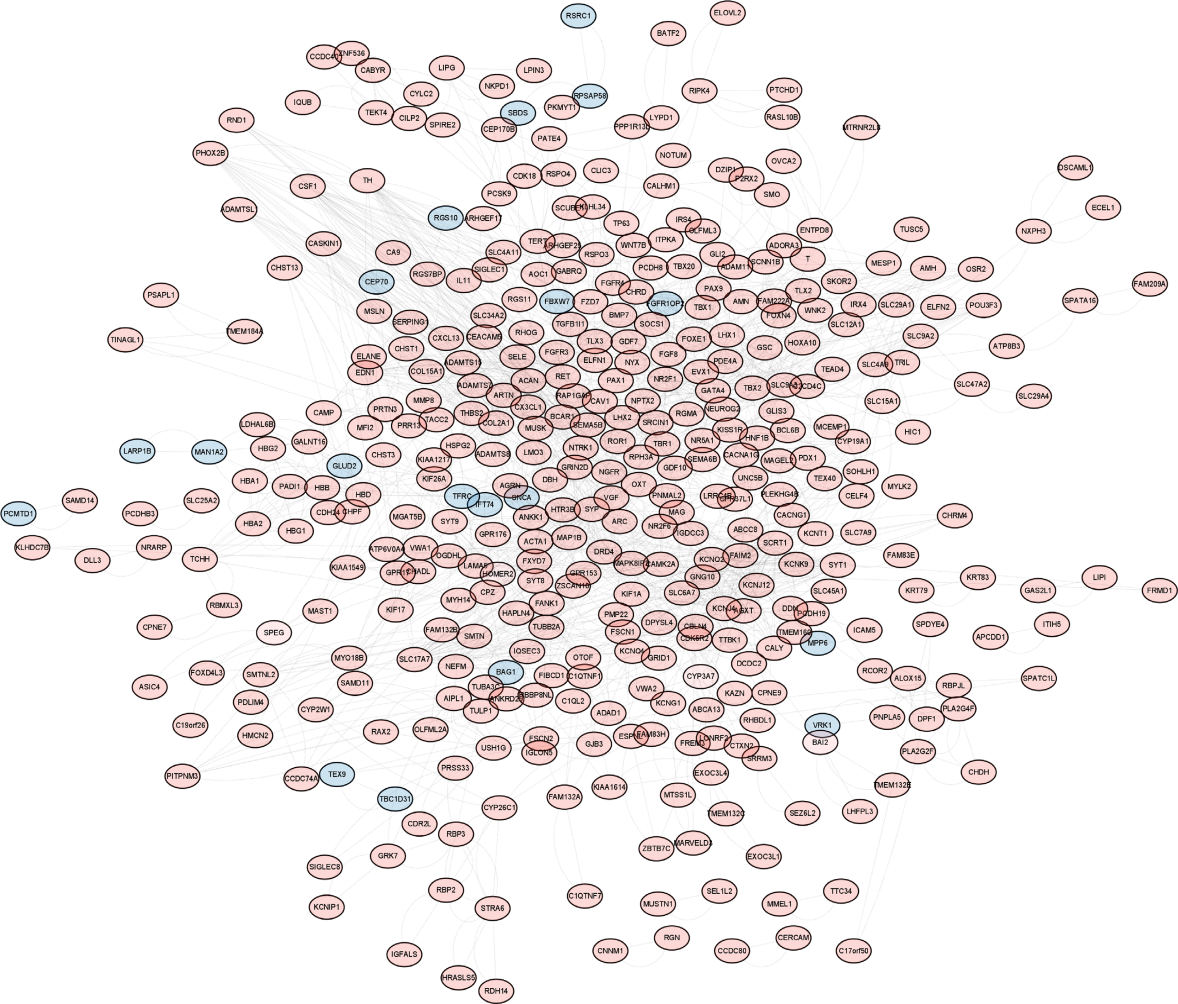
Up- and downregulation relationships of differentially expressed genes. Display of interacting proteins by Cytoscape. Red represents upregulated genes, and blue represents downregulated genes.

Dataset GSE184050 was downloaded from the GEO database. A total of 618 DEGs were obtained by differential analysis and displayed using volcano plots (**Figure 2**). Some genes were selected for heatmap display based on their log2FC values (**Figure 3**), the protein−protein self-test interactions of DEGs were displayed using PPI (**Figure 4**), and 576 genes that were upregulated and 42 genes that were downregulated were visualized by Cytoscape (**Figure 5**).

### Identification of 9 hub genes

**Figure 6:**
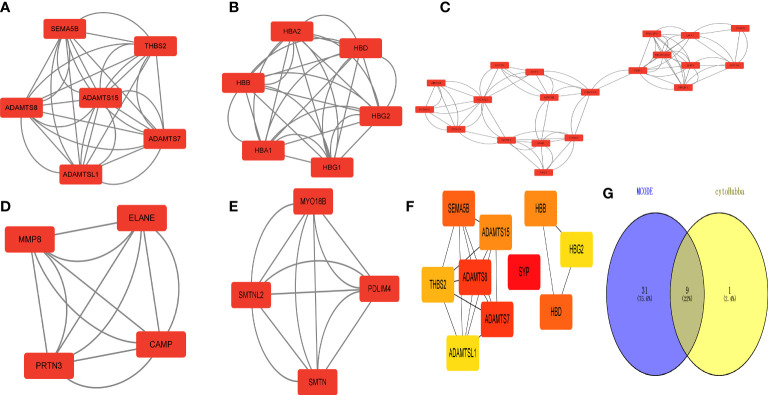
Identification of 618 differentially expressed genes (DEGs) in type 2 diabetes. Hub gene screening. **(A–E)** MCODE plug-in scores > 4 for key modules. **(F)** Key hub genes found by the Hubba plug-in. **(G)** Key genes found by both methods taken from the intersection of the Venn diagram.

The MCODE plug-in identified 5 important modules (**Figures 6A–E**). Then, the top 10 network hub genes were identified using the Hubba plug-in, with the hub genes creating a separate network (**Figure 6F**). The key genes found by the 2 methods were intersected to obtain 9 hub genes, which were displayed using a Venn diagram (**Figure 6G**).

### Enrichment analysis of differentially expressed genes in type 2 diabetes

**Figure 7:**
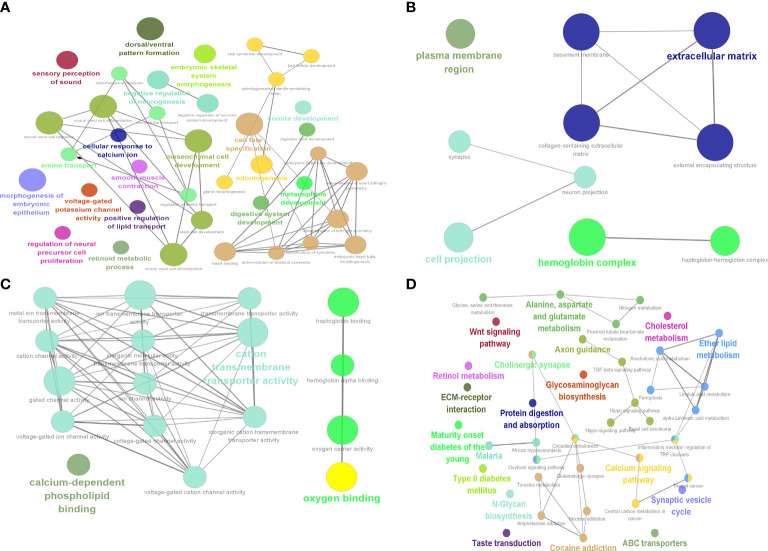
Identification of 9 hub genes. Results of DEG enrichment analysis. **(A)** BP display diagram. **(B)** CC display diagram. **(C)** MF display diagram. **(D)** KEGG display diagram. The color represents the P value, and the size of the circle represents the number of genes. BP, biological processes; CC, cellular components; MF, molecular functions; KEGG, Kyoto Encyclopedia of Genes and Genomes.

GO and KEGG enrichment results for DEGs showed that BP (**Figure 7A**) enrichment included positive regulation of lipid transport, CC (**Figure 7B**) enrichment included hemoglobin complex and basement membrane, MF (**Figure 7C**) enrichment included oxygen binding and hemoglobin alpha binding, and KEGG (**Figure 7D**) was enriched for linoleic acid metabolism and ferroptosis.

### Gene set enrichment analysis validation

**Figure 8:**
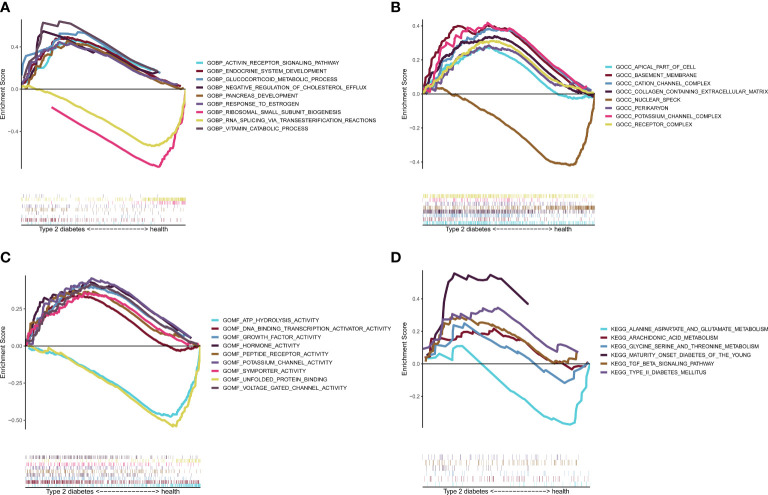
Gene set enrichment analysis (GSEA) validation. GSEA enrichment verification analysis. **(A)** BP display. **(B)** CC display. **(C)** MF display. **(D)** KEGG display. BP, biological processes; CC, cellular components; MF, molecular functions; KEGG, Kyoto Encyclopedia of Genes and Genomes.

The GSEA enrichment results showed that BP (**Figure 8A**) enrichment included ENDOCRINE_SYSTEM_DEVELOPMENT, CC (**Figure 8B**) enrichment included basement_membrane, MF (**Figure 8C**) enrichment included ATP_HYDROLYSIS_ACTIVITY, and KEGG (**Figure 8D**) enrichment included TYPE_II_DIABETES_MELLITUS and TGF_BETA_SIGNALING_PATHWAY, which verified six potential signaling pathways in T2D.

### Construction of the celastrol pharmacophore target network

**Figure 9:**
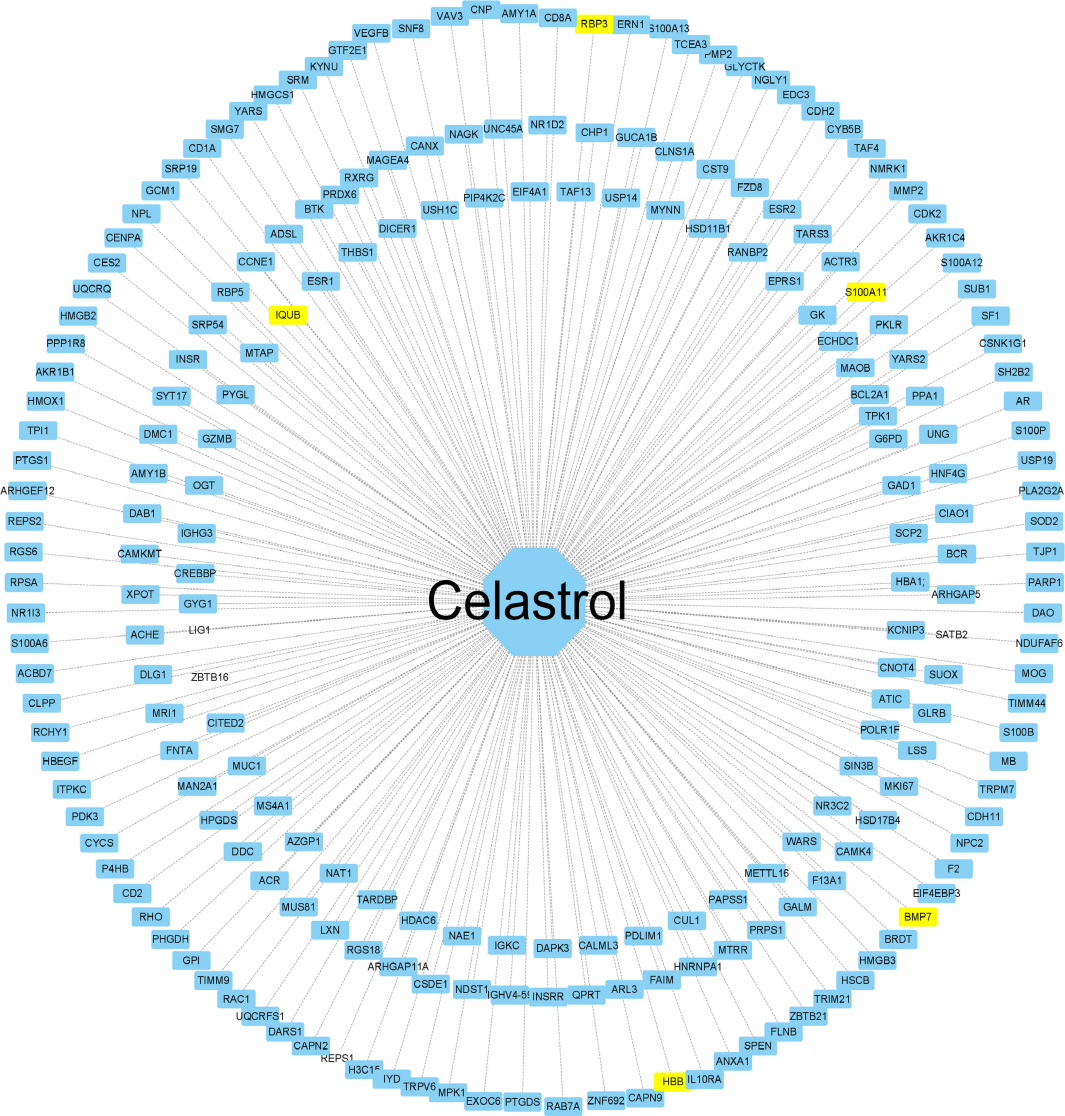
Construction of the celastrol pharmacophore target network. “Drug-target” network. The drug celastrol is in the middle, and the targets of the network are annotated targets of successful pharmacophore genes (yellow indicates the drug and the corresponding disease target gene). The middle line is the target relationship line.

The 2D structure of celastrol was successfully retrieved from the PubChem database, and the PharmMapper database predicted the top 600 pharmacophore target genes based on the combined score. Finally, 228 genes were successfully annotated in the UniProt annotation database using the “Homo sapiens” species as the criterion. The annotated genes were intersected with the DEGs to obtain the target genes for celastrol, including S100A11, RBP3, HBB, BMP7 and IQUB, and the annotated genes were then imported into Cytoscape software to complete the network construction of celastrol and the target genes (**Figure 9**).

### Celastrol pharmacophore target enrichment analysis and identification of signaling pathways for therapy

**Figure 10:**
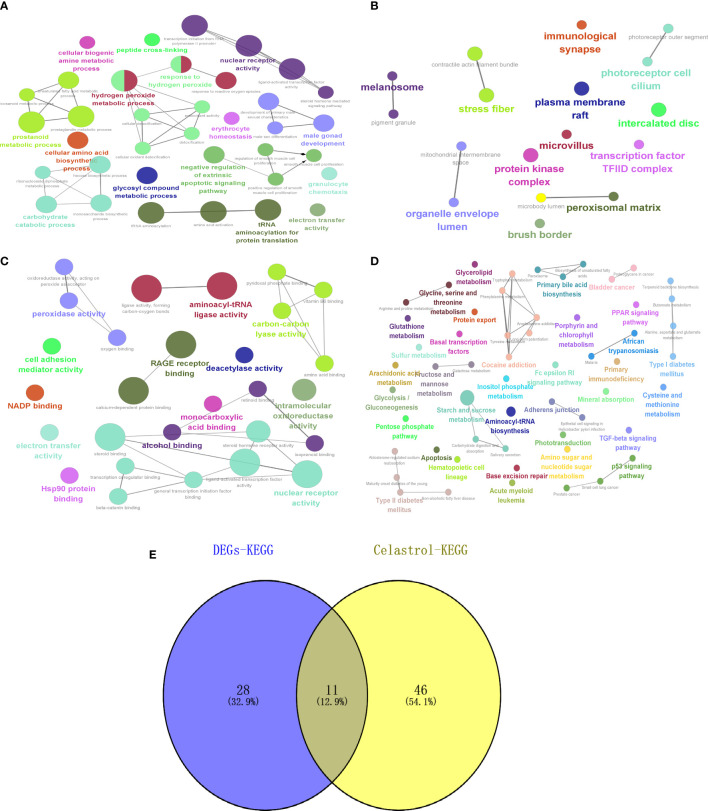
Celastrol pharmacophore target enrichment analysis and identification of signaling pathways for therapy. Celastrol enrichment analysis and analysis of potential signaling pathways for treatment. **(A)** Circle diagram of BP. **(B)** Circle diagram of CC. **(C)** Circle diagram of MF. **(D)** Circle diagram of KEGG. **(E)** Venn diagram of “DEGs-KEGG” and “Celastrol-KEGG”. The darker the color is, the smaller the p value, and the size of the circle represents the number of genes. BP, biological processes; CC, cellular components; MF, molecular functions; KEGG, Kyoto Encyclopedia of Genes and Genomes.

GO and KEGG enrichment analysis of the drug targets of celastrol showed that BP (**Figure 10A**) was enriched in glycosyl compound metabolic process, CC (**Figure 10B**) was enriched in mitochondrial intermembrane space and transcription factor, MF (**Figure 10C**) enrichment included intramolecular oxidoreductase activity and oxidoreductase activity, and KEGG (**Figure 10D**) enrichment included the type II diabetes mellitus and TGF-beta signaling pathways. Then, the intersections of the signaling pathways enriched with DEGs were shown by a Venn diagram, and six potential signaling pathways for celastrol treatment of T2D were obtained (**Figure 10E**). These pathways included maturity onset diabetes of the young, type II diabetes mellitus, African trypanosomiasis, malaria, alanine, aspartate and glutamate metabolism, glycine, serine and threonine metabolism, TGF-beta signaling pathway, tyrosine metabolism, cocaine addiction, amphetamine addiction and arachidonic acid metabolism.

### AlphaFold2 prediction of the protein structure of celastrol therapeutic targets

**Figure 11:**
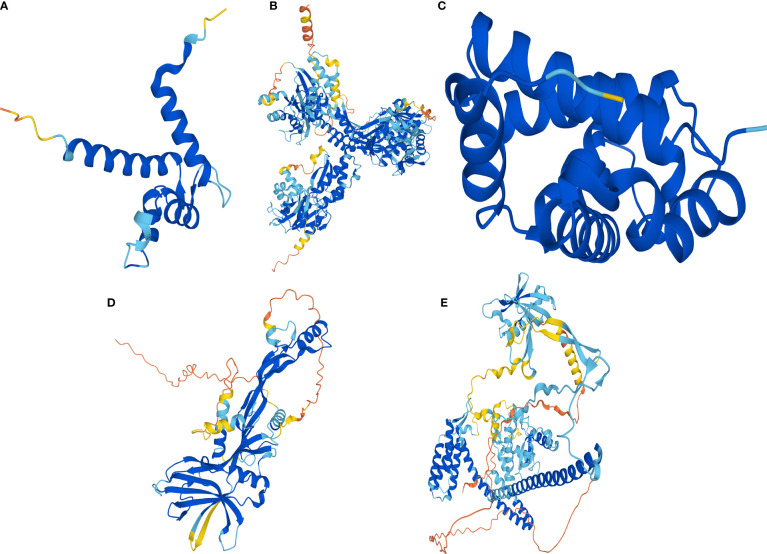
AlphaFold2 prediction of the protein structure of celastrol therapeutic targets. Protein structures of the therapeutic targets predicted by the artificial intelligence AlphaFold2. **(A)** Predicted protein structure of S100A11. **(B)** Predicted protein structure of RBP3. **(C)** Predicted protein structure of HBB. **(D)** Predicted protein structure of BMP7. **(E)** Predicted protein structure of IQUB.

Human protein numbers for celastrol therapeutic targets were found in the UniProt online database and included P10745-RBP3, P18075-BMP7, P31949-S100A11, P68871-HBB and Q8NA54-IQUB. Then, AlphaFold2 was used to predict their protein structures, and the protein structures of S100A11 (**Figure 11A**), RBP3 (**Figure 11B**), HBB (**Figure 11C**), BMP7 (**Figure 11D**) and IQUB (**Figure 11E**) were obtained.

### Molecular docking validation of celastrol and therapeutic targets

**Figure 12:**
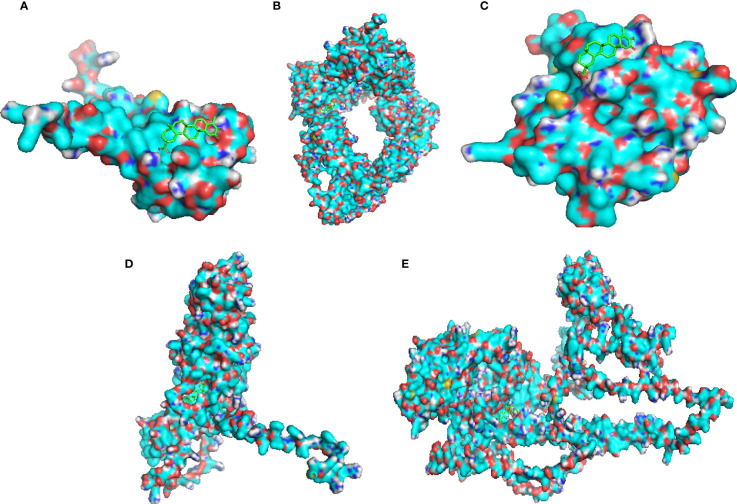
Molecular docking validation of celastrol and therapeutic targets. Molecular docking of the small-molecule ligand celastrol to the protein receptor of the target gene. **(A)** Molecular docking display diagram of S100A11. **(B)** Molecular docking display diagram of RBP3. **(C)** Molecular docking display diagram of HBB. **(D)** Molecular docking display diagram of BMP7. **(E)** Molecular docking demonstration of IQUB. The green structure is the small-molecule ligand for celastrol, and the blue structure is the protein receptor for the target gene.

The protein structures of the celastrol target genes were successfully validated by molecular docking, and the results are shown in **Table 1**. Those with the lowest free energy were selected for separate presentation. The molecular docking results for S100A11 (**Figure 12A**), RBP3 (**Figure 12B**), HBB (**Figure 12C**), BMP7 (**Figure 12D**) and IQUB (**Figure 12E**) were selected to show the lowest free energy.

**Table 1:** Binding energy values of celastrol to therapeutic targets.

Target	drug	Binding energy/(kcal/mol)
**BMP7**	**Celastrol**	**-11.0**
**HBB**	**Celastrol**	**-8.3**
**IQUB**	**Celastrol**	**-8.0**
**RBP3**	**Celastrol**	**-7.5**
**S100A11**	**Celastrol**	**-7.4**

## Discussion

T2D is a chronic disease with a high prevalence and a wide range of complications, the main ones being obesity, hypertension, atherosclerosis and osteoporosis. Lifestyle interventions can improve the symptoms of T2D (32) and can improve the symptoms of T2D. However, according to the FDA and EMA in the US and Europe, there are no recommended drugs for T2D prevention. Celastrol is a natural herbal chemical that prevents T2D (33) and improves insulin resistance (34) through a variety of pathways and has played controlling roles in many chronic diseases (35). It also has a protective effect against type 2 diabetes combined with NAFLD in mice (36). In our study, we used a bioinformatic approach to identify the hub genes and key signaling pathways involved in the development of T2D and used network pharmacology and the artificial intelligence AlphaFold2 to try to elucidate the potential regulatory mechanisms of celastrol through the hub genes and key signaling pathways for the treatment of T2D.

By bioinformatics analysis, we identified nine relatively important hub genes, including ADAMTS15, ADAMTS7, ADAMTSL1, SEMA5B, ADAMTS8, THBS2, HBB, HBD and HBG2. THBS2 increases susceptibility to T2D in diabetic patients (37) and is significantly associated with T2D prevalence (38). HBB increases the risk of having T2D (39). HBD is detected in various systematic tests for diabetes (40). HBG2 is upregulated in diabetic retinopathy (41). The ADAMTS15, ADAMTS7, ADAMTSL1, SEMA5B and ADAMTS8 genes should be further investigated.

The enrichment analysis of DEGs revealed a total of 48 enriched signaling pathways, of which 6 were validated as significant by GSEA, including maturity onset diabetes of the young, type II diabetes mellitus, alanine, aspartate and glutamate metabolism, glycine, serine and threonine metabolism, TGF-beta signaling pathway and arachidonic acid metabolism. Maturity-onset diabetes of the young and type II diabetes mellitus are both disease signaling pathways of diabetes. Glycine, serine and threonine metabolism and the TGF-beta signaling pathway have been discussed in previous reports. The TGF-beta signaling pathway has also been verified as key signaling pathway of T2D by GSEA (42), and the activation of the TGF-beta signaling pathway can alleviate hepatic steatosis and fibrosis in T2D (43). Arachidonic acid metabolism is significantly related to Qi deficiency in T2D (44) and can improve the symptoms of T2D (45). The signaling pathways of alanine, aspartate and glutamate metabolism need further study.

Using pharmacophore target analysis in a network pharmacology approach, we identified five potential target genes for celastrol in the treatment of type 2 diabetes, including RBP3, BMP7, S100A11, HBB and IQUB. BMP7 gene therapy counteracts insulin resistance and obesity (46). S100A11 expression is reduced during the treatment of impaired glucose tolerance, further reducing the prevalence of T2D (47). Of these, HBB is a hub gene and is described in the GeneCards database as a protein-coding gene for HBB. Diseases associated with HBB include sickle cell anemia and beta-thalassemia, the dominant inclusion type. Patients with transfusion-dependent beta-thalassemia are at high risk of developing diabetes mellitus (48). Sickle cell traits may increase the risk of developing T2D-related complications (49). IQUB and RBP3 are subject to further validation. Enrichment analysis was then performed with 228 target genes, resulting in 57 signaling pathways, which intersected with the 48 signaling pathways enriched by DEGs to obtain 11 signaling pathways for celastrol treatment, six of which were validated in GSEA, with the remaining five including African trypanosomiasis, malaria, tyrosine metabolism, cocaine addiction and amphetamine addiction. T2D increases the risk of infection by malaria (50), and products of malaria can reverse T2D (51). Tyrosine metabolism is a characteristic of oxidative stress as a complication of T2D (52). African trypanosomiasis, cocaine addiction and amphetamine addiction need to be further verified.

Finally, we used the artificial intelligence AlphaFold2 to predict the human protein structures of the five target genes, which were successfully validated by molecular docking with celastrol. The advent of artificial intelligence has accelerated the progress of scientific research, especially with AlphaFold2, which accelerates the development of new drugs by predicting protein structures with high accuracy rates, providing a powerful tool for new drug research in chronic diseases. This research combines bioinformatics, network pharmacology and artificial intelligence approaches to provide a new research strategy for the development of therapeutic drugs for diseases.

## Conclusion

In this study, we used a bioinformatics approach to identify hub genes and signaling pathways in the development of T2D. Celastrol may prevent and treat T2D through key target genes, such as HBB, as well as signaling pathways, such as the TGF-beta signaling pathway and type II diabetes mellitus, providing a new strategy for the prevention and treatment of T2D with celastrol. The key targets and potential signaling pathways for celastrol in the treatment of T2D were further analyzed using network pharmacology.

## Data availability statement

The original contributions presented in the study are included in the article/supplementary material. Further inquiries can be directed to the corresponding author.

## Author contributions

MW and YZ: design and initiation of the studies, monitoring of data quality, data analysis and interpretation, and preparation and editing of the manuscript. MW and YZ: data acquisition. YZ: experimental operation and data acquisition. MW and YZ: research concept and design and research initiation. All authors contributed to the article and approved the submitted version.

## Funding

The present study was supported by the National Natural Science Foundation of China (Grant No. 81772383) and funded by the Outstanding Leaders Training Program of Pudong Health Bureau of Shanghai (Grant No. PWRL2019-01).

## Conflict of interest

The authors declare that the research was conducted in the absence of any commercial or financial relationships that could be construed as a potential conflict of interest.

## Publisher’s note

All claims expressed in this article are solely those of the authors and do not necessarily represent those of their affiliated organizations, or those of the publisher, the editors and the reviewers. Any product that may be evaluated in this article, or claim that may be made by its manufacturer, is not guaranteed or endorsed by the publisher.

## Abbreviations

GEO, Gene Expression Omnibus; T2D, Type 2 Diabetes; BP, biological processes; CC, cellular components; MF, molecular functions; KEGG, Kyoto Encyclopedia of Genes and Genomes; PPI, protein−protein interaction; DEGs, differentially expressed genes; GO, gene ontology; GSEA, gene set enrichment analysis; Log2FC, log 2 fold change.
